# Supplementary material for: Yeasts Prefer Daycares and Molds Prefer Private Homes
Source: Microb Ecol. 2025 Feb 20;88(1):7. doi: 10.1007/s00248-025-02505-4 (PMC11842513; doi:10.1007/s00248-025-02505-4)
Supplement: Supplementary file 1 — Supplementary file1 (PDF 239 KB) [file 248_2025_2505_MOESM1_ESM.pdf]

Supplementary Material - Figures and Tables

Journal name: *Microbial Ecology*

Article title: *Yeasts prefer daycares and molds prefer private homes*

Authors: Håvard Kauserud[a]#*, Pedro M. Martin-Sanchez[a,b]#*, Eva Lena Estensmo[a,c], Synnøve Botnen[d], Luis Morgado[e], Sundy Maurice[a], Klaus Høiland[a], Inger Skrede[a]

[a]University of Oslo, Department of Biosciences, Section for Genetics and Evolutionary Biology (Evogene), Oslo, Norway.
[b]Instituto de Recursos Naturales y Agrobiología de Sevilla (IRNAS-CSIC), Seville, Spain.
[c]Norwegian Veterinary Institute, Ås, Norway.
[d]Oslo Metropolitan University, Oslo, Norway.
[e]Naturalis Biodiversity Center, Leiden, Netherlands.

#Håvard Kauserud and Pedro M. Martin-Sanchez contributed equally to this work. Author order was determined on the basis of the alphabet.

*Corresponding authors:
Håvard Kauserud: haavarka@ibv.uio.no;
Pedro M Martin-Sanchez: pmartin@irnase.csic.es

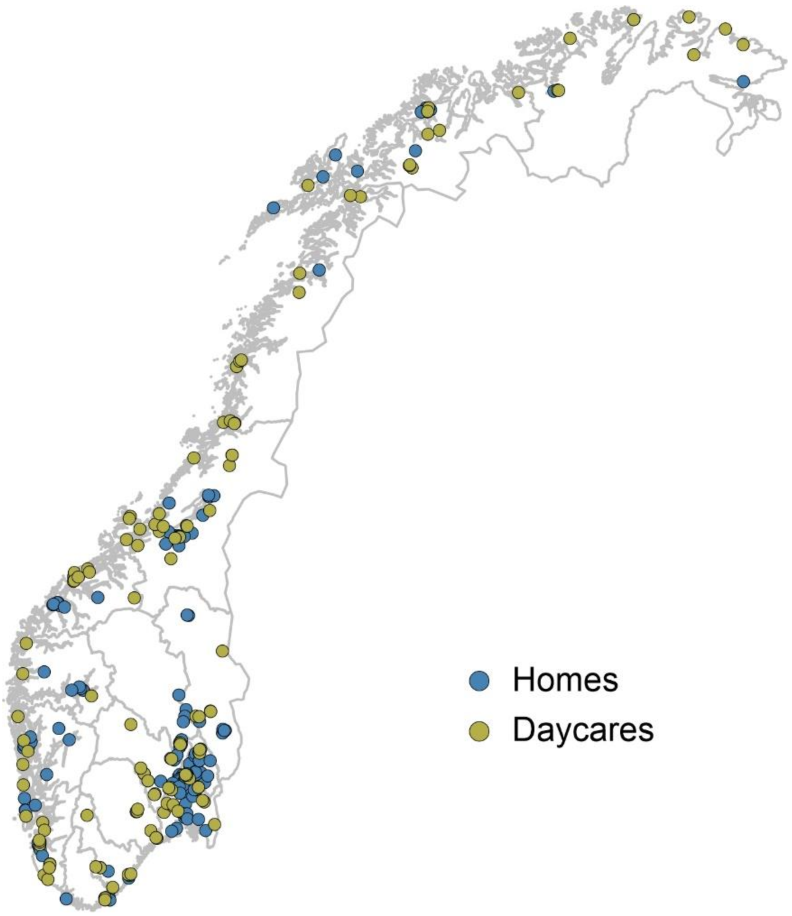

Figure S1. Location and distribution of the private homes (214) and daycares (123) selected throughout Norway for this study.

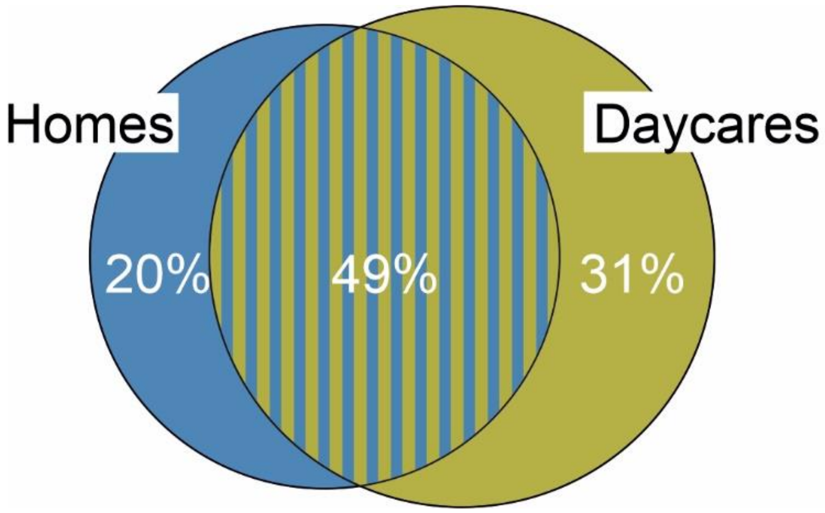

Figure S2. Overlap of indoor dust mycobiomes (8,181 OTUs) between homes and daycares.

Supplementary Tables

Table S1. Variables analyzed in this study

Variables (as in metadata file)	Description (if needed)	Numeric vs. Categorical	Categories (as in metadata file)
dataset	homes vs. daycares	C	house / kindergarten
indoor_outdoor		C	indoor / outdoor
room		C	outside / central / bathroom
latitude	latitudes (58.1-71.03)	N	
longitude	longitudes (5-29.7)	N	
municipality	origin municipality in Norway	C	139 municipalities
area		C	rural / urban
construction_year	construction years (1746 to 2018)	N	
building_type		C	single_building /collection_of_houses
natural_ventilation		C	yes / no
mechanical_ventilation		C	yes / no
balanced_ventilation		C	yes / no
children	number of children (0-176)	N	
people	number of people (1-179)	N	
pets	presence of pets	C	yes / no
water_damage	reported water damage	C	yes / no
pests	reported pests including mice, rats and grey silverfish	C	yes / no
mean_temp	annual mean temperature BIO1 (WorldClim)	N	
temp_seasonality	temperature seasonality BIO4 (WorldClim)	N	
mean_temp_driest	mean temperature of the driest quarter BIO9 (WorldClim)	N	
mean_temp_warmest	mean temperature of the warmest quarter BIO10 (WorldClim)	N	
mean_temp_coldest	mean temperature of the coldest quarter BIO11 (WorldClim)	N	
annual_prec	annual precipitation BIO12 (WorldClim)	N	
growing_season_length	Growing season length[a]	N	

swe_4	snow water equivalent in April[a]	N	
sca_2	snow-covered area in February[a]	N	
total_insolation	potential incoming solar radiation[a]	N	
ar50	land cover AR 50[a]	C	developed area / agricultural area / forest / barren land / bog and fen / fresh water
bedrock_nutrient	bedrock nutrient[a]	C	poor / average / rich

[a] Source: Horvath, P., Halvorsen, R., Stordal, F., Tallaksen, L. M., Tang, H., & Bryn, A. (2019). Distribution modelling of vegetation types based on area-frame survey data. Applied Vegetation Science, 22, 547–560. https://doi.org/10.1111/avsc.12451

Table S2. OTUs identified as indoor indicator fungal species (IndVal > 50%; $p < 0.05$) for homes and daycares using the R package *indicspecies* (De Cáceres & Legendre, 2009).

Phylum	Order	Species	Relative read abundance (%)	Occurrence[¶] (# of 839 indoor samples)	Ind Val (%)
**Home indicators**					
Ascomycota	Helotiales	*Botrytis cinerea*	1.77	645	94
Ascomycota	Eurotiales	*Penicillium olsonii*	0.95	514	91
Ascomycota	Eurotiales	*Penicillium* sp.	0.50	510	87
Ascomycota	Pleosporales	*Alternaria arborescens*	0.33	438	84
Basidiomycota	Agaricales	*Strobilurus esculentus*	0.77	465	82
Basidiomycota	Polyporales	*Fomitopsis pinicola*	0.68	388	81
Ascomycota	Capnodiales	*Cladosporium sphaerospermum*	0.15	388	76
Ascomycota	Eurotiales	*Aspergillus versicolor*	0.27	416	74
Ascomycota	Eurotiales	*Aspergillus niger*	0.62	357	74
Basidiomycota	Hymenochaetales	*Resinicium bicolor*	0.21	374	73
Ascomycota	Eurotiales	*Penicillium carneum*	0.16	318	70
Basidiomycota	Polyporales	*Fomes fomentarius*	0.26	292	69
Unidentified fungus	unidentified	unidentified	0.15	322	64
Ascomycota	Eurotiales	*Penicillium digitatum*	0.26	266	63
Basidiomycota	Agaricales	*Strobilurus stephanocystis*	0.17	246	62
Ascomycota	Eurotiales	*Aspergillus fumigatus*	0.13	241	61

Ascomycota	Eurotiales	*Penicillium* sp.	0.22	292	59
Ascomycota	Eurotiales	*Aspergillus penicillioides*	0.11	241	58
Basidiomycota	Polyporales	*Cerrena unicolor*	0.08	260	58
Ascomycota	Capnodiales	*Cladosporium* sp.	0.13	191	58
Ascomycota	Xylariales	*Annulohypoxylon multiforme*	0.03	182	53
Basidiomycota	Russulales	*Heterobasidion annosum*	0.05	204	53
Basidiomycota	Polyporales	*Antrodia* sp.	0.04	161	53
Ascomycota	Pleosporales	*Neoascochyta exitialis*	0.06	206	51
**Daycare indicators**					
Basidiomycota	Hymenochaetales	*Trichaptum abietinum*	2.28	478	80
Basidiomycota	Filobasidiales	*Filobasidium magnum*	0.63	526	70
Mucoromycota	Mucorales	*Mucor plumbeus*	2.33	437	77
Basidiomycota	Pucciniales	*Melampsoridium betulinum*	1.04	436	74
Basidiomycota	Hymenochaetales	*Trichaptum fuscoviolaceum*	4.77	245	74
Ascomycota	Xylariales	*Truncatella angustata*	0.24	368	73
Basidiomycota	Holtermanniales	*Holtermanniella festucosa*	0.14	398	71
Basidiomycota	Ustilaginales	*Ustilago nunavutica*	0.51	343	68
Basidiomycota	Tremellales	*Cryptococcus uniguttulatus*	0.22	240	64
Ascomycota	Saccharomycetales	*Cyberlindnera* sp.	0.22	266	64
Mucoromycota	Mucorales	*Mucor hiemalis*	0.15	279	61
Basidiomycota	Filobasidiales	*Filobasidium* sp.	0.06	256	60
Basidiomycota	Leucosporidiales	*Leucosporidium scottii*	0.06	229	55
Basidiomycota	Polyporales	*Amylocystis lapponica*	0.17	132	55
Basidiomycota	Urocystidales	*Urocystis agropyri*	0.28	222	54
Basidiomycota	Cystofilobasidiales	*Cystofilobasidiaceae*	0.09	202	53
Ascomycota	Saccharomycetales	*Saccharomyces* sp.	0.06	198	52
